# The Effect of *Lactobacillus casei* 32G on the Mouse Cecum Microbiota and Innate Immune Response Is Dose and Time Dependent

**DOI:** 10.1371/journal.pone.0145784

**Published:** 2015-12-29

**Authors:** Busra Aktas, Travis J. De Wolfe, Kanokwan Tandee, Nasia Safdar, Benjamin J. Darien, James L. Steele

**Affiliations:** 1 Department of Food Science, University of Wisconsin, Madison, WI, United States of America; 2 Food Science and Technology, Maejo University, Chiangmai, Thailand; 3 Infectious Diseases Division, Department of Medicine, University of Wisconsin, Madison, WI, United States of America; 4 William S. Middleton Veterans Affairs Hospital, Madison, WI, United States of America; 5 Animal Health and Biomedical Sciences, University of Wisconsin, Madison, WI, United States of America; Charité, Campus Benjamin Franklin, GERMANY

## Abstract

Lactobacilli have been associated with a variety of immunomodulatory effects and some of these effects have been related to changes in gastrointestinal microbiota. However, the relationship between probiotic dose, time since probiotic consumption, changes in the microbiota, and immune system requires further investigation. The objective of this study was to determine if the effect of *Lactobacillus casei* 32G on the murine gastrointestinal microbiota and immune function are dose and time dependent. Mice were fed *L*. *casei* 32G at doses of 10^6^, 10^7^, or 10^8^ CFU/day/mouse for seven days and were sacrificed 0.5h, 3.5h, 12h, or 24h after the last administration. The ileum tissue and the cecal content were collected for immune profiling by qPCR and microbiota analysis, respectively. The time required for *L*. *casei* 32G to reach the cecum was monitored by qPCR and the 32G bolus reaches the cecum 3.5h after the last administration. *L*. *casei* 32G altered the cecal microbiota with the predominance of *Lachnospiraceae* IS, and *Oscillospira* decreasing significantly (p < 0.05) in the mice receiving 10^8^ CFU/mouse 32G relative to the control mice, while a significant (p < 0.05) increase was observed in the prevalence of lactobacilli. The lactobacilli that increased were determined to be a commensal lactobacilli. Interestingly, no significant difference in the overall microbiota composition, regardless of 32G doses, was observed at the 12h time point. A likely explanation for this observation is the level of feed derived-nutrients resulting from the 12h light/dark cycle. 32G results in consistent increases in Clec2h expression and reductions in TLR-2, alpha-defensins, and lysozyme. Changes in expression of these components of the innate immune system are one possible explanation for the observed changes in the cecal microbiota. Additionally, 32G administration was observed to alter the expression of cytokines (IL-10rb and TNF-α) in a manner consistent with an anti-inflammatory response.

## Introduction

The human gastrointestinal tract hosts over 10^14^ cells made up of 500 to 1000 bacterial species collectively known as the human microbiom. The human microbiome is believed to play an important role in health and disease [1,2]. The gut microbiota is believed to serve as an organ which takes a part in many physiological and homeostatic functions [3–6]. It has been shown that a well balanced bidirectional interaction between the microbiota and the immune system exist. This complex relationship begins at birth, and has been shown to have a fundamental role in development and maturation of the immune system, but at the same time the immune system plays a role in shaping the microbiota composition and functions that will last a lifetime [3,7,8]. Disfunction in the balance has been linked to many diseases including inflammatory diseases such as inflammatory bowel disease (IBD) [9,10]. There are several interventions likely to modify the indigenous intestinal microbiota, including diet and antibiotics. Probiotics are one of the diet related interventions that have been shown to alter the microbial composition of the gut. Probiotics are live microorganisms, which when administered in adequate amounts, confer a health benefit on the host [11]. Probiotics have potential to influence gut dysbiosis and they have also been shown to help maintain immune health[12,13]. For example, *C*. *difficile* infection is believed to be caused by a collapse of the microbial community in the gut after an antibiotic assault that imbalances the microbiota [14,15]. Many patients return to the hospital with a recurrence of *C*. *difficile* infections. Probiotics could be a useful approach in restoring and supporting the “good” community in the gut, helping to restore balance the intestinal microbiota. A diverse and rapidly expanding set of health benefits have been ascribed to probiotics including: improved ability to tolerate lactose; reduction in gastrointestinal pathogens; reduction in colorectal cancer; decrease in incidence of cold and flu; and a reduction in the symptoms associated with the inflammation-related disorders, such as ulcerative colitis [16–18]. Probiotics come from a variety of genera, including *Lactobacillus*, *Bifidobacterium*, *Propionibacterium*, *Escherichia*, and *Saccharomyces*; however, the most commonly used strains are from the genus’ *Lactobacillus* and *Bifidobacterium* [19].

*Lactobacillus* are a component of the gut microbiota, are one of the major genera commonly used as probiotics and numerous health benefits have been associated with their use as probiotics [17,19–22] While a detailed mechanistic understanding of the probiotic effects of lactobacilli is lacking, it has commonly been thought that changes in the host gut microbiota are one reason for these beneficial health outcomes [23,24]. For example, Bruzzese et al. linked the consumption of *Lactobacillus* GG with improved cystic fibrosis and changes in the gut microbiota [24]. However, conflicting results concerning the linkage of changes in the gut microbiota and probiotic-related health impacts have also recently been reported [25]. A 2014 study by Aoki et al. demonstrated that *L*. *casei* Shirota helps to improve aberrant bowel movements in patients with gastrectomies. Interestingly, no change in the subjects’ fecal microbiota was detected [22]. Similarly Eloe-Fadrosh et al. demonstrated that consumption of *L*. *rhamnosus* GG administration to 12 health individuals, 65 to 80 years old, had an anti-inflammatory effect and linked this outcome not with changes in the composition of the gut microbiota, but rather with changes in activity of the commensal microbiota [23]. Understanding the seemingly contradictory results from these studies will require an understanding of the mechanism(s) by which probiotics alter the composition of the gut microbiota

*Lactobacillus casei* is a commonly utilized probiotic species, with more than 32 thousands servings per day consumed worldwide through dairy products like Yakult and DanActive [26]. *L*. *casei* are Gram (+), facultatively anaerobic, industrially important lactic acid bacteria that have been isolated from a variety of diverse habitats, including fermented dairy products (i.e., cheese) and plant materials (i.e., wine, pickle, and silage,), as well as the gastrointestinal (GI) tract, and oral cavity of humans and animals [27]. The *L*. *casei* species has been analyzed by Multilocus Sequence Typing (MLST) and determined to diverge into three major lineages approximately 1.5 million years ago [28]. Previously, our research group screened four different *L*. *casei* strains for their ability to survive the GI tract passage, adhere to ileum epithelium, and influence the intestinal microbiota in a piglet and a murine model [29]. *L*. *casei* 32G was found to survive passage through the GI tract and modify the ileum microbiota in both animal models. In this study, we monitored the abundance of the fed strain at different time points in the cecum and examined the impact of *L*. *casei* 32G dose and time since probiotic consumption on the mouse commensal cecal microbiota using 16S rRNA sequencing. Due to the well-described relationship between the gut microbiota and host immunity, we also examined the relationship between the probiotic strain, gut microbiota, and immune function.

## Material and Methods

### Bacterial strain

*L*. *casei* 32G was maintained at -80°C in MRS broth (BD Difco, Sparks, MD) with 25% (v/v) glycerol (Sigma-Aldrich, St. Louis, MO). Working cultures were prepared from frozen stocks by two sequential transfers in MRS broth and incubations were conducted statically at 37°C for 24 h and 18 h, respectively. The culture at early stationary phase was harvested by centrifugation at 5,000 rpm for 10 min at room temperature. The pellet was re-suspended in 0.85% NaCl (w/v) and the optical density at 600 nm (OD_600_) determined. A volume of washed cells (based upon the OD_600_) sufficient to yield a 5 ml cell suspension with an OD_600_ of 6.0 was harvested by centrifugation at 5,000 rpm and washed with 5 ml of 0.85% NaCl. The resulting pellet was suspended in 5 ml of 0.85% NaCl to obtain a final concentration of 10^9^ CFU/ml. The culture was serially diluted in 0.85% NaCl to reach concentrations of 10^8^ and 10^7^ CFU/ml. The final culture solution that was kept at 4°C until the administration was enumerated daily on MRS agar to confirm the dose administered to the mice.

### Animals

All procedures involving mice were conducted under the protocol #V01548 approved by the Animal Care and Use Committee of the University of Wisconsin-Madison. Healthy, male C57BL/6 mice aged 8 weeks were obtained from Jackson Laboratories (Bar Harbor, ME) and group housed at University of Wisconsin-Madison Animal Health and Biomedical Science facility. Housing conditions were controlled at 25°C, 20–44% relative humidity with a 12 h light/dark cycle. Mice were fed *ad libitum* water and mouse chow (Harlan Teklad 7964 rodent diet, Madison, WI) throughout the study. The animals (n:96) included in this study were divided into 5 groups; each group (n:30) was administered daily 100 μl of either 0.85% NaCl (control), 10^7^, 10^8^, or 10^9^ CFU/ml of *L*. *casei* 32G by oral gavage for seven days. Therefore, the delivered doses were 10^6^ (low dose), 10^7^ (medium dose), and 10^8^ (high dose) CFU/day/mouse. *L*. *casei* 32G was fed for 7 days to simulate long-term daily consumption of a probiotic, as is typically suggested for probiotic consumption.

### Sample collection

Six mice from each group were euthanized by CO_2_ asphyxiation at 0.5h, 3.5h, 12h, 24h and 72h after administration of the last probiotic dose to evaluate the effect of time and immediately after euthanization the intestinal tract was removed for analysis. The cecum content was collected and the samples were immediately put on ice, and then frozen at -20°C until processed for microbial DNA extraction. Approximately 2 cm-tissue from the distal ileum was collected for RNA isolation and preserved in RNAlater (Ambion, Carlsbad, CA) overnight at 4°C. After the overnight treatment, the samples were stored at -80°C until processing.

### Determination of lactic acid content in the cecum content

The amount of D- and L-lactic acid in the cecum digesta was determined using D-Lactic acid/L-lactic acid kit from R-Biopharm (Darmstadt, Switzerland). A 0.9 ml sample of digesta was centrifuged at 20,000 ×g for 1 h and the supernatant was recovered. Proteins present in the supernatant were precipitated using the Carrez clarification reagent (85 mM K4[Fe(CN)6] and 250 mM ZnSO4) as described for the D-Lactic acid/L-lactic acid kit and the pH adjusted to 8.0 using 1N NaOH. Subsequently, D- and L-lactate were quantified as directed by the supplier, except that the total volume of the assay was decreased from 3 ml to 600 μl, while maintaining the proportions described in the manufacturer’s instructions for each reagent.

### DNA extraction

The cecum digesta was homogenized in 1.5ml of PBS and total DNA from 200 μl of the homogenate was isolated using the QIAamp DNA Stool Mini Kit (Qiagen Sciences, MD) with modifications to the manufacturer’s instructions. These modifications included an initial mechanical cell disruption step by inclusion of 0.1 mm glass beads (Sigma-Aldrich) followed by exposure to six 1 min beating at maximum speed in a Mini-beadbeater-96 (Biospec Products, Inc., Bartlesville, OK) with intervals of 2 min on ice. Subsequently, a heat treatment step was performed for 5 min at 95°C. The DNA was further purified by phenol:chloroform:isoamyl alcohol (25:24:1, pH 8) extraction, phase separation using Phase Lock Gels (5 PRIME) and ethanol precipitation using pellet paint co-precipitant (EMD Millipore). DNA was quantified by Qubit® 2.0 Fluorometer (Invitrogen, Carlsbad, CA). Extracted DNA was used to perform 16S rRNA sequencing and *L*. *casei* 32G detection by qPCR.

### Ion Torrent PGM Sequencing and Microbiota Analysis

Partial 16S rRNA sequences were determined on a 318 v2 chip using the Ion Torrent Personal Genome Machine System at University of Wisconsin-Madison, Biotechnology Center. Briefly, the V1-V2 region was amplified using forward primers that contained a sample-specific bar-code with an Ion A adapter and a key sequence, while the associated reverse primer contained a truncated P1 (trP1) adapter. The sequence of these primers were: forward (8FM—5'–*CCA TCT CAT CCC TGC GTG TCT CCG AC* *T CAG* BBB BBB BBB BBB BAG AGT TTG ATC MTG GCT CAG—3') with the Ion A adapter in italics, the key sequence in italics and underlined, the 13 bp bar code designated as Bs, and the 16S primer sequence in capital letters; reverse (357R - 5'–*CCT CTC TAT GGG CAG TCG GTG AT*C TGC TGC CTY CCG TA- 3') with the trP1 adapter in italics and the 16S primer sequence in capital letters. All PCR reactions were quality-controlled for amplicon saturation by gel electrophoresis. Equal quantities of each of the amplicons were pooled and purified using AxyPrep Mag PCR beads (Corning, Inc.). The resulting products were quantified using PicoGreen (Invitrogen) and Qubit fluorometer (Invitrogen) before sequencing. The sequences were deposited in the NCBI BioProject database with the study identification number SRP062166. The data processing pipeline removed low-quality reads that: 1) did not completely match the PCR primer and barcode; 2) were shorter than 300 bp or longer than 400 bp in length; or 3) had an average quality score <22. Data analysis was performed in QIIME 1.8 framework [30]. Operational Taxonomic Units (OTUs) were chosen with QIIME picking OTU workflow based upon sequence similarity with a 97% similarity threshold. Taxonomic identities were assigned using greengenes version 13_5 [31].

*Lactobacillus* sequences extracted from the microbiota dataset were blasted against NCBI database to identify the species with highest identity.

### RNA isolation and Gene Expression Analysis

Tissue samples from the distal small intestine were homogenized in UltraPure guanidine isothiocyanate solution (Invitrogen) using a tissue grinder with a smooth pestle (Thomas Scientific, Swedesboro, NJ). RNA was isolated using PureLink RNA mini kit (Invitrogen) as recommended by the supplier. Concentrations and purity of RNA samples were determined with a NanoDrop 2000 spectrophotometer (Thermo Scientific, Waltham, MA) and the integrity was checked by the control reaction included in the Bio-Rad prime PCR assay. Total RNA was treated with DNase I (Invitrogen) to remove DNA contamination and subsequently converted into cDNA using iScriptTM cDNA synthesis kit (Bio-Rad, Hercules, CA) according to manufacturer’s protocol. qPCR was performed using the primers shown at Table 1 and the customized 96-well prime PCR assays (Bio-rad) were used to screen 29 different genes of interest. SsoFast™ EvaGreen® Supermix (Bio-Rad) was used under the following conditions: initial denaturation at 95°C for 2 min, followed by 40 cycles of 5 sec at 95°C and 30 sec at 60°C. Data were acquired in the final step at 95°C for 5 sec and melting curves (65 to 95°C) were generated at the end for each set of primers. Gene expression was normalized to β-actin and relative gene expression was calculated by 2^-ΔΔCt^ method [36].

**Table 1:** Primers used in qPCR analysis.

Gene^*^	Forward	Reverse	Reference
*Defa-rs1c*	5'-CACCACCCAAGCTCCAAATACACAG-3'	5'-ATCGTGAGGACCAAAAGCAAATGG-3'	[32]
*Defcr1*	5’-TCAAGAGGCTGCAAAGGAAGAGAAC-3’	5’-TGGTCTCCATGTTCAGCGACAGC-3’	[32]
*Defcr4*	5’-CCAGGGGAAGATGACCAGGCTG-3’	5’-TGCAGCGACGATTTCTACAAAGGC-3’	[32]
*Lyz1-2*	5’-GCCAAGGTCTACAATCGTTGTGAGTTG-3’	5’-CAGTCAGCCAGCTTGACACCACG-3’	[32]
*Pla2g2*	5’-AGGATTCCCCCAAGGATGCCAC-3’	5’-CAGCCGTTTCTGACAGGAGTTCTGG-3’	[32]
*sPLA2-IIA*	5’-ACAGGTCCAAGGGAACATTG-3’	5’-TCTGGTTTGCAGAACAGGTG-3’	[33]
*Occludin*	5’-CCCTGACCACTATGAAACAG-3’	5’-TTGATCTGAAGTGATAGGTG-3’	[34]
*ZO-1*	5’-CCTAAGACCTGTAACCATCT-3’	5’-CTGATAGA- TATCTGGCTCCT-3’	[34]
*ZO-2*	5’-CTAGACCCCCAGAGCCCCAGAAA-3’	5’-TCGCAGGAGTCCACGCATACAAG-3’	[35]

* The common names: cryptdin-related sequence (*Defa-rs1c*); cryptdin-1 (*Defcr1*); cryptdin-4 (*Defcr4*); Lysozyme (*Lyz1*); zonula occludens (*ZO*).

### *Lactobacillus casei* 32G detection by qPCR

Total DNA extracted from cecum digesta was used to detect *L*. *casei* 32G, the fed microorganism. A deletion in the 32G *dltX* gene was identified and a strain specific primer to the junction on the deletion was designed. The primer set was checked for sequence similarity by the BLAST (NCBI). The sequences of the primers are as follows; 5′- AAG TGA ACA GAC ACG CAT CG -3′ and 5′- AAC GCC TGT CAG CTT CAT CT-3′. The primers were tested by qPCR with an annealing temperature gradient. The following conditions were applied: initial denaturation at 95°C for 30 sec, followed by 40 cycles of 5 sec at 95°C and 5 sec at 57°C. A melting curve was generated at the end for the primer set. Samples considered positive for the presence of 32G yielded a single sharp melting curve. A matrix based standard curve was created. The cecum content suspended with PBS was spiked with 10^8^, 10^7^, 10^6^, 10^5^, 10^4^ and 10^2^ CFU/ml of *L*. *casei* 32G culture, which was confirmed by enumerating on MRS, and DNA was extracted from each sample. DNA was amplified by qPCR in triplicate.

### Statistical analysis

For microbiota data, the statistical difference between treatments was examined using the Monte-Carlo test in package ade4 [37] of R 2.14.0 [38] as described by de Carcer et al [39]. The values of zero were replaced with the detection limit, which is determined by the ratio of one to the lowest read number in the data set. The dominant genera that increased or decreased in abundance were identified by running the correspondence analysis provided in the package ade4 of R 2.14.0 as described by de Carcer et al [39]. The Benjamini-Hochberg procedure was applied to control the false discovery rate. Statistical difference for relative gene expression was assessed with the Wilcoxon rank sum test (Mann–Whitney test) using JMP version 10 (SAS Institute Inc., Cary, NC) and was presented as mean ± SEM. Statistical difference was determined at a P value of 0.05 or less.

## Results and Discussion

### Detection of *Lactobacillus casei* 32G in mice after oral administration

**Fig 1:**
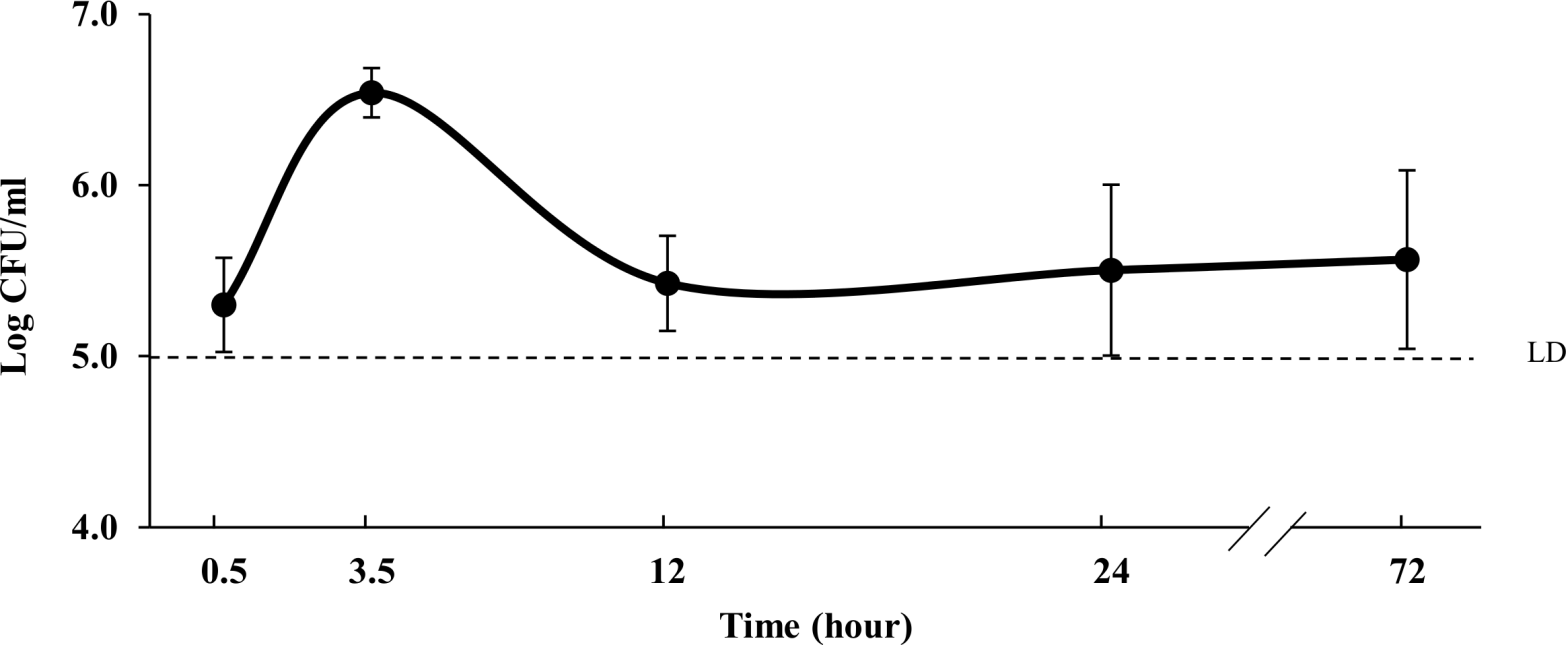
*L*. *casei* 32G detection in cecum digesta of mouse fed with 10^8^ CFU/mouse at 0.5h, 3.5h, 12h, 24h, and 72h after last administration. **(n:6/time point).** LD, limit of detection.

To initiate research on the mechanism(s) by which *L*. *casei* 32G influences cecal microbiota composition and modulates the immune system, transit through the murine GI tract was followed using qPCR. A qPCR primer set specific to 32G was designed based on the publically available genome on NCBI. To determine the limit of detection and establish a standard curve, cecum content obtained from control mice were spiked with known quantities of *L*. *casei* 32G. 32G was detected in samples containing 10^5^ CFU/ml or greater quantities of 32G, indicating that this was the limit of detection of the method (S1 Fig). To evaluate GI transit of 32G, cecum samples were collected from mice fed with the high dose (10^8^ CFU/day) of *L*. *casei* 32G at different time points after the last administration. *L*. *casei* 32G was detected in all time points at levels ranging from 5.3 to 6.5 log CFU/ml, with the highest concentration being observed at the at the 3.5h time point (Fig 1). It is important to note that this analysis does not provide information concerning the viability of the organisms detected. These results are similar to those described by Daniel et al., who examined transit of *L*. *plantarum* using bioluminescence imaging. Their results indicate that *L*. *plantarum* took about 90 min to reach the cecum and localized there until 4h after the last administration [40]. *L*. *planatarum* was also monitored previously by Marco et al. by enumerating lactobacilli in mouse feces and by measuring the level of 16S rRNA in gut compartments after a single oral administration; however, *L*. *plantarum* was not selectively monitored [41]. To determine if 32G could be detected from all three dose levels, qPCR analysis was conducted with the 3.5h cecum samples from all three doses. *L*. *casei* 32G was detected in 0, 2, and 4 of the 4 samples examined from the 10^6^, 10^7^, and 10^8^ CFU/day samples, respectively (S1 Table). These results demonstrate that only mice fed a high dose of 32G contained greater than 10^5^ CFU/ml of 32G in their cecal contents and that 32G was present at the highest level 3.5 h after administration.

### *Lactobacillus casei* 32G alters the cecal microbiota composition depending on dose and time

The interaction between the commensal GI microbiota and immune system is an area of intense research interest [4,12]. This interest has resulted in a rapid increase in the number of investigations on the impact of probiotics on the GI microbiota; however, only rarely has effect of dose and time since probiotic consumption been examined. In this study, we examined the impact of *L*. *casei* 32G dose and time since probiotic consumption on the mouse commensal cecal microbiota using 16S rRNA sequencing. Ion Torrent PGM sequencing of cecal content was conducted to assess the influence of *L*. *casei* 32G dose and time on the mice GI microbiota. The sequencing resulted in a total of 2,239,643 filtered reads from 96 mice cecum digesta samples; the number of reads varied from 7,535 to 42,559 with an average of 18,664 reads per sample. After the taxonomic status of each read was assigned, 9 phyla, 16 classes, 29 orders, 48 families, 72 genera were identified. To assess whether sufficient sequence reads had been collected to accurately determine the diversity of organisms present, shannon and chao1 index were examined; the results of this analysis are presented in S2 Fig. These results indicate that sufficient sequence reads were obtained to accurately describe the diversity present in these samples. Additionally a principal coordinate analysis (PCA) plots using weighted Unifrac distances were generated to provide a visual overview of gut microbial dynamics in response to administration of *L*. *casei* 32G (S3 Fig).

**Fig 2:**
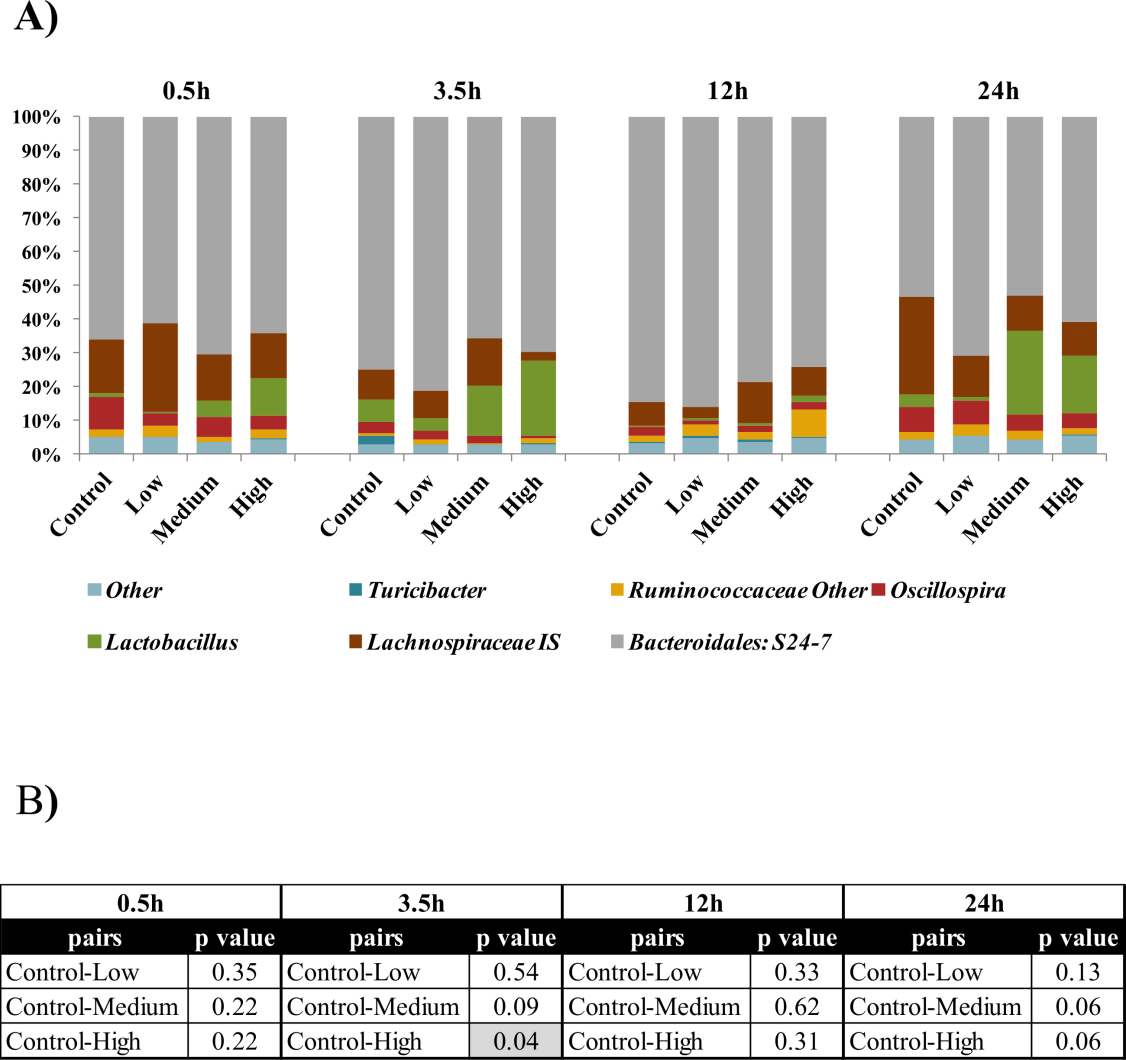
Comparison of predominant genera in the microbiota of mice cecums sorted based on time. (A) The major microbial communities of mouse cecum content at genus level in the control group and *L*. *casei* 32G groups; 10^6^ CFU/ mouse (low), 10^7^ CFU/ mouse (medium) and 10^8^ CFU/ mouse (high), at 0.5, 3.5, 12, and 24 h after the last administration. Only genera with over 5% of the total bacteria are presented (n: 6 for each bar). (B) Pair wise comparison of each group. Statistical p-values were assessed using a Monte-Carlo test with 10000 replicates. The values with p≤0.05 are highlighted.

The cecum microbiota of the mice fed with 32G at 10^6^ (low), 10^7^ (medium) and 10^8^ CFU/day (high) doses at different time points after the last administration were compared with the control mice at genus level. As determined by Monte-Carlo analysis, the overall microbiota of the 32G-fed mice did not differ significantly (p < 0.05) from the controls for the low and the medium dose. However, the overall microbiota differed significantly (p < 0.05) at the 3.5h time points for high dose, (Fig 2). The mouse-to-mouse variation in the cecum microbiota is presented in S4 Fig. The dominant genera that increased or decreased in abundance were identified by correspondence analysis. When the samples were compared at the genus level by correspondence analysis, significant (p < 0.05) changes were observed between all samples and their controls, with a 12, 23, and 25 changes at the genus level for the 10^6^, 10^7^, and 10^8^ CFU/day doses, respectively (Table 2). These results demonstrate that 32G dose has a significant (p < 0.05) influence on the composition of the cecum microbiota.

**Table 2:** Bacterial genera detected^a^ in cecum content of mice administered saline (control) or *Lactobacillus casei* 32G once per day by oral gavage sorted based on time.

Taxon	Percentage (mean ± SE)^b^ ^c^															
	0.5h				3.5h				12h				24h			
	Control	Low	Medium	High	Control	Low	Medium	High	Control	Low	Medium	High	Control	Low	Medium	High
*S24-7 IS*	66.2±15.2	61.4±27.7	70.4±13.0	64.3±25.3	75.0±15.8	81.3±17.5	65.9±5.9	69.7±15.6	84.6±10.3	86.2±5.9	78.8±20.3	**74.1**±**14.0**	53.6±22.0	70.8±20.7	52.9±31.2	60.9±18.1
*Lachnospiraceae IS*	15.9±9.9	26.1±25.0	13.8±9.6	13.2±8.5	8.9±7.2	8.0±10.2	13.9±8.3	**2.7**±**1.6**	7.1±6.0	**3.3**±**2.5**	12.2±14.6	8.8±8.4	28.8±19.7	**12.3**±**11.3**	**10.7**±**12.2**	**10.2**±**6.7**
*Oscillospira*	10.0±4.7	**3.8**±**1.6**	**5.9**±**3.9**	**4.3**±**4.1**	3.3±3.0	2.7±3.6	2.0±1.3	**0.8**±**0.6**	2.4±2.8	1.0±0.6	2.1±2.7	1.9±1.6	7.2±3.3	7.1±6.2	4.9±4.9	**4.2**±**3.1**
*Ruminococcaceae Other*	2.1±1.2	3.2±2.3	**1.2**±**0.8**	2.4±1.9	0.6±0.3	**1.4**±**0.6**	0.5±0.2	**1.5**±**1.0**	2.0±1.4	3.3±1.9	2.0±2.1	**8.3**±**4.1**	2.3±1.4	3.2±1.9	2.6±2.5	2.2±0.9
*Ruminococcus*	1.3±0.6	1.1±0.7	**0.4**±**0.2**	1.2±1.4	0.7±0.4	0.5±0.4	**0.2**±**0.1**	0.7±0.8	1.0±0.5	**1.9**±**1.4**	0.7±0.8	1.2±0.7	0.8±0.3	1.0±0.3	0.6±0.5	1.4±1.2
*Clostridia Other*	1.2±0.8	1.0±0.6	**0.4**±**0.4**	0.7±0.7	0.3±0.1	0.6±0.6	0.2±0.1	0.2±0.1	0.5±0.5	0.6±0.3	0.3±0.2	0.6±0.5	0.9±0.3	0.8±1.2	0.7±0.7	0.8±0.6
*Lactobacillus*	0.9±1.6	0.5±0.9	**5.0**±**4.6**	**11.0**±**16.4**	6.8±7.7	3.6±6.9	**15.0**±**9.6**	**22.2**±**14.6**	0.4±0.5	0.6±0.9	0.6±0.4	1.9±2.7	3.7±4.3	1.1±1.0	**24.8**±**17.3**	**16.9**±**19.5**
*Lachnospiraceae Other*	0.9±0.4	0.9±0.5	**0.4**±**0.2**	0.7±0.6	0.3±0.2	0.3±0.4	0.2±0.1	0.2±0.1	0.4±0.5	0.7±0.8	0.3±0.2	0.6±0.5	0.8±0.4	1.1±1.5	0.5±0.5	0.8±0.4
*Clostridiales Other*	0.6±0.3	0.6±0.4	0.5±0.4	0.4±0.4	0.1±0.1	0.3±0.3	0.2±0.1	0.1±0.1	0.3±0.3	0.2±0.1	0.3±0.2	**0.6**±**0.5**	0.6±0.3	0.5±0.3	0.6±0.8	0.4±0.2
*Ruminococcaceae IS*	0.2±0.1	0.3±0.4	**0.9**±**0.6**	0.7±0.9	0.3±0.2	0.2±0.3	**0.6**±**0.3**	**0.8**±**0.4**	0.2±0.1	0.6±0.7	**1.0**±**0.6**	**0.8**±**0.5**	0.2±0.1	0.4±0.2	**0.4**±**0.3**	**0.5**±**0.2**
*Firmicutes Other*	0.2±0.1	0.1±0.2	0.2±0.1	0.2±0.2	0.2±0.1	0.2±0.2	0.2±0.1	0.2±0.1	0.2±0.2	0.2±0.1	0.2±0.1	0.1±0.1	0.2±0.1	**0.4**±**0.3**	**0.4**±**0.2**	**0.4**±**0.2**
*Bacteria Other*	0.2±0.0	**0.1**±**0.1**	**0.3**±**0.1**	0.2±0.1	0.2±0.1	0.2±0.1	**0.4**±**0.2**	0.3±0.1	0.2±0.1	0.1±0.0	0.2±0.1	**0.4**±**0.2**	0.2±0.1	0.2±0.1	**0.3**±**0.1**	**0.5**±**0.1**
*Erysipelotrichaceae IS*	0.1±0.1	0.2±0.2	0.1±0.1	**0.0**±**0.0**	0.0±0.0	0.1±0.0	**0.1**±**0.1**	0.0±0.0	0.1±0.1	0.1±0.1	**0.2**±**0.2**	0.0±0.0	0.0±0.0	**0.1**±**0.1**	**0.1**±**0.0**	**0.1**±**0.0**
*Turicibacter*	0.0±0.1	0.1±0.2	0.0±0.0	**0.3**±**0.4**	2.7±2.6	**0.2**±**0.2**	**0.1**±**0.2**	**0.4**±**0.7**	0.4±0.5	0.8±0.9	0.8±1.1	0.4±0.5	0.1±0.1	**0.3**±**0.3**	**0.0**±**0.0**	0.0±0.1
*Coprobacillaceae IS*	0.0±0.0	0.0±0.0	0.0±0.0	0.0±0.0	0.0±0.0	**0.1**±**0.1**	0.0±0.0	0.0±0.0	0.0±0.0	0.0±0.0	0.0±0.0	0.0±0.0	0.0±0.0	**0.2**±**0.2**	0.0±0.0	**0.0**±**0.0**
*Adlercreutzia*	BQL	0.1±0.1	0.2±0.1	0.0±0.0	0.2±0.5	0.1±0.2	0.3±0.2	0.1±0.0	0.1±0.2	0.1±0.2	0.2±0.1	**0.0**±**0.0**	0.1±0.0	0.2±0.3	0.2±0.2	0.1±0.1
*Coriobacteriales IS*	BQL	BQL	BQL	BQL	0.0±0.0	BQL	BQL	BQL	BQL	BQL	BQL	BQL	0.0±0.0	0.0±0.0	BQL	**0.2**±**0.2**
**Number of alterations** ^**d**^	-	2	8	4	-	3	6	6	-	2	2	6	-	5	7	9

^a^Only genera that were present at ≥1% in a sample are included in this table.

^b^The detection limit was 0.00003 and this value was used to calculate the p-value.

^c^Genera that differ from control within each group are shown in bold (p≤0.05).

^d^The number of genera that differed from the control for that treatment.

*IS*: *Incertae Sedis*.

BQL: Below quantifiable limit.

The most abundant genus found in the cecum content of all of the samples was *Bacteroidales S24*-7 *Incertae Sedis* (*IS*), a poorly studied genus, which accounted for 53%-86% of the total microbiota. The dominant genera (> 3% of the total microbiota) in rank order of the cecum microbiota in the control mice were *S24*-7 *IS*, *Lachnospiraceae IS*, *Oscillospira* and *Lactobacillus*, together these genera comprise 93% of the total microbiota (Table 2). Our qPCR results demonstrated that 32G reaches the cecum 3.5h after administration; therefore, the influence of the 32G administration on the mouse cecum microbiota was evaluated with the 3.5h samples. The high dose samples were selected as this dose resulted in the largest number of alterations to the cecum microbiota. The predominance of *Lachnospiraceae IS*, and *Oscillospira* decreased significantly (p < 0.05) in the mice receiving 10^8^ CFU/mouse 32G relative to the control mice; in contrast, a significant (p < 0.05) increase was observed in the prevalence of *Lactobacillus* (Table 2, Fig 2). *Lachnospiraceae IS*, a butyrate producing superfamily, decreased from 8.9% to 2.7% [42,43]. A decrease in *Lachnospiraceae IS* was also observed in our previous study done on 32G fed piglets [29]. *Lachnospiraceae* are highly abundant in human gut [43] and are known to decrease the severity of colitis and the degree of *Clostridium difficile* colonization [44,45]. Ravussin et al. reported that the greatest difference in cecal microbiota between diet induced obese mice and the control mice was the level of *Lachnospiraceae*, which were found at a much higher level in obese mice [46]. *Oscillospira* is the other genus that decreased (from 3.3% to 0.8%) at the 3.5h time point as a result of 32G administration. *Oscillospira*, an uncultured group, are normally found in rumen of cattle and sheep, have been detected in human large intestine [47], and have been shown to be depleted in obese patients [48]. *Lactobacillus* increased (from 6.8% to 22.2%) significantly (p < 0.05) in high dose mice at 3.5h, in comparison to their control mice [49]. In IBS patients, commensal *Lactobacillus* are depleted; similarly stress has been observed to result in decreases in lactobacilli in animals [5]. Previous studies have observed that *Lachnospiraceae* and *Oscillospira* prevalence typically increase or decrease together and that *Lactobacillus* typically changes in opposite direction [50,51], as was observed in this study. There are a number of possible explanations for these observations including: microbe-microbe interactions; microbe-host interactions; and the impact of diet on the composition of the microbiota.

Although this study was not designed to evaluate the influence of the 12h dark/light photoperiodic cycle on the composition of the cecum microbiota, the lack of significant differences in the overall microbiota composition, regardless of 32G dose, of all samples at 12h was compelling (Figs 2 and S5, S2 Table). Previous researchers have demonstrated that C57BL/6 mice, the same strain used in this study, had their highest food consumption during early dark cycle when fed *ad libitum* [52,53]. Based on the 24-hour Zeitgeber time units (ZT), where the lights are turned on as ZT0 and off at ZT12, our 12h time point corresponds to ZT13, which is four hours after increased food intake starts. At this time point the microbiota composition likely reflects the presence of a low level of feed-derived nutrients, as time is required for both transit of the food and changes in microbial composition. This suggests that the light/dark cycle has a significant impact on the composition of the cecum microbiota, likely due to the level of feed derived-nutrients [54], and hence must be taken into consideration when designing experiments that follow microbiota composition.

### The increase in prevalence of *Lactobacillus* in the intestinal microbiota was not directly due to the fed microorganism, *Lactobacillus casei* 32G

**Fig 3:**
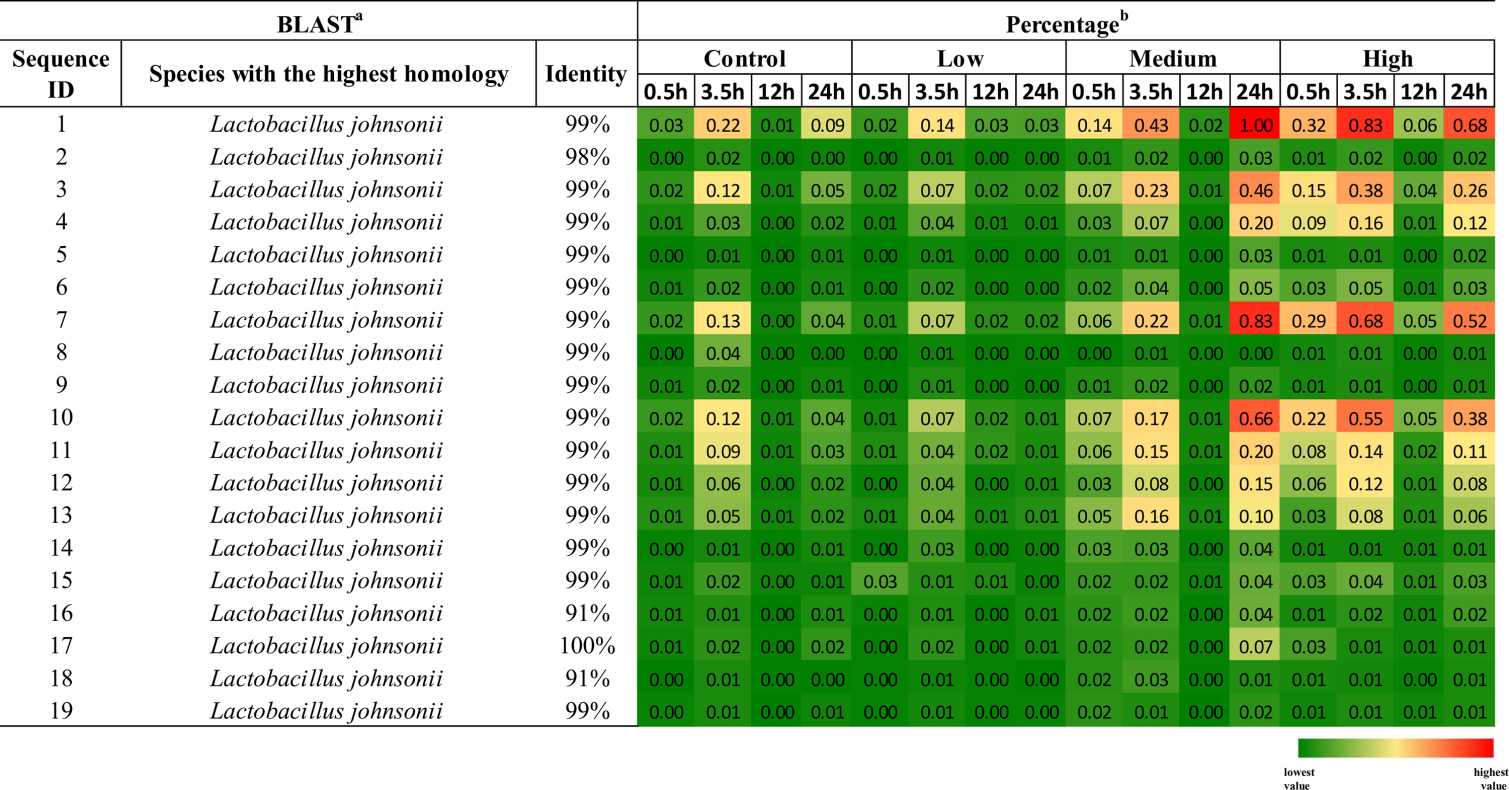
The species of *Lactobacillus* detected in the cecums of *L*. *casei* fed mice. The species of *Lactobacillus* with the highest 16S rRNA sequence identity to those present in the cecums of *L*. *casei* fed mice *Lactobacillus* sequences detected were blasted against NCBI database to identify the species with the highest identity (a). The heat map depicts the relative abundance of *Lactobacillus* sequences detected in the cecums of *L*. *casei* fed mice. The percentage was calculated from the average values for each sample (n:6/time point) and a graded color scale is utilized to visualize the relative abundance (b).

*Lactobacillus* was observed to increase at all time points other than 12h in mice fed 10^7^ or 10^8^ CFU *L*. *casei* 32G. This increase could either be due directly to the presence of 32G or indirectly by 32G altering the conditions present in the cecum, thereby allowing the commensal lactobacilli to increase in prevalence. To investigate whether the lactobacilli that increased in prevalence in the intestinal microbiota were *L*. *casei*, we extracted the representative genera sequences from the QIIME pipeline. Nineteen distinct *Lactobacillus* sequences were present more than 500 times in the overall dataset, these sequences were blasted against the NCBI database and the species with the highest homology are presented in Fig 3. In all cases, *Lactobacillis johnsonii* was the species with the highest homology to the sequences present in these samples, although the sequence identity was relatively low (91%) for two of the *Lactobacillus* sequences. *L*. *casei* sequence identity with these reads varied from 80 to 84% and there were always at least 10 species with higher identity than *L*. *casei*. These findings suggest that the *Lactobacillus* which increased in prevalence were *L*. *johnsonii*, not *L*. *casei* 32G. *L*. *johnsonii* are commensal lactobacilli present in the murine gut [55,56]. One explanation of these results is that feeding of *L*. *casei* 32G at 10^7^ and 10^8^ CFU/mouse daily altered the environment present in the cecum such that the growth of commensal lactobacilli was favored.

### Lactic acid concentration and pH of the cecum content

Intestinal pH is one of the factors involved in modification of gut microbiota [1,39,57,58]. The pH of the cecum digesta obtained from each group of mice is presented in S3 Table. Interestingly, there was no significant difference in the mice fed with *L*. *casei* 32G, when compared to the control mice, at any dose at 3.5h time point, when the 32G bolus passes the cecum. Considering that lactic acid is the primary metabolic end product of *L*. *casei* carbohydrate fermentation [59], we measured the lactate concentration in the cecum samples collected from the mice subjected to the high dose 32G at 3.5h. There were also no significant differences in the D-lactate, L-lactate or total lactate concentrations (data not shown). Moreover, there was a slight increase in the pH of the samples from the medium (0.5 and 24h) and high dose (0.5h) which could be result of products by commensals in the gut. These results suggest that neither pH nor lactic acid had a major role in the observed 32G associated changes in cecal microbiota.

### Intestinal innate immune profile associated with *L*. *casei* 32G administration

**Fig 4:**
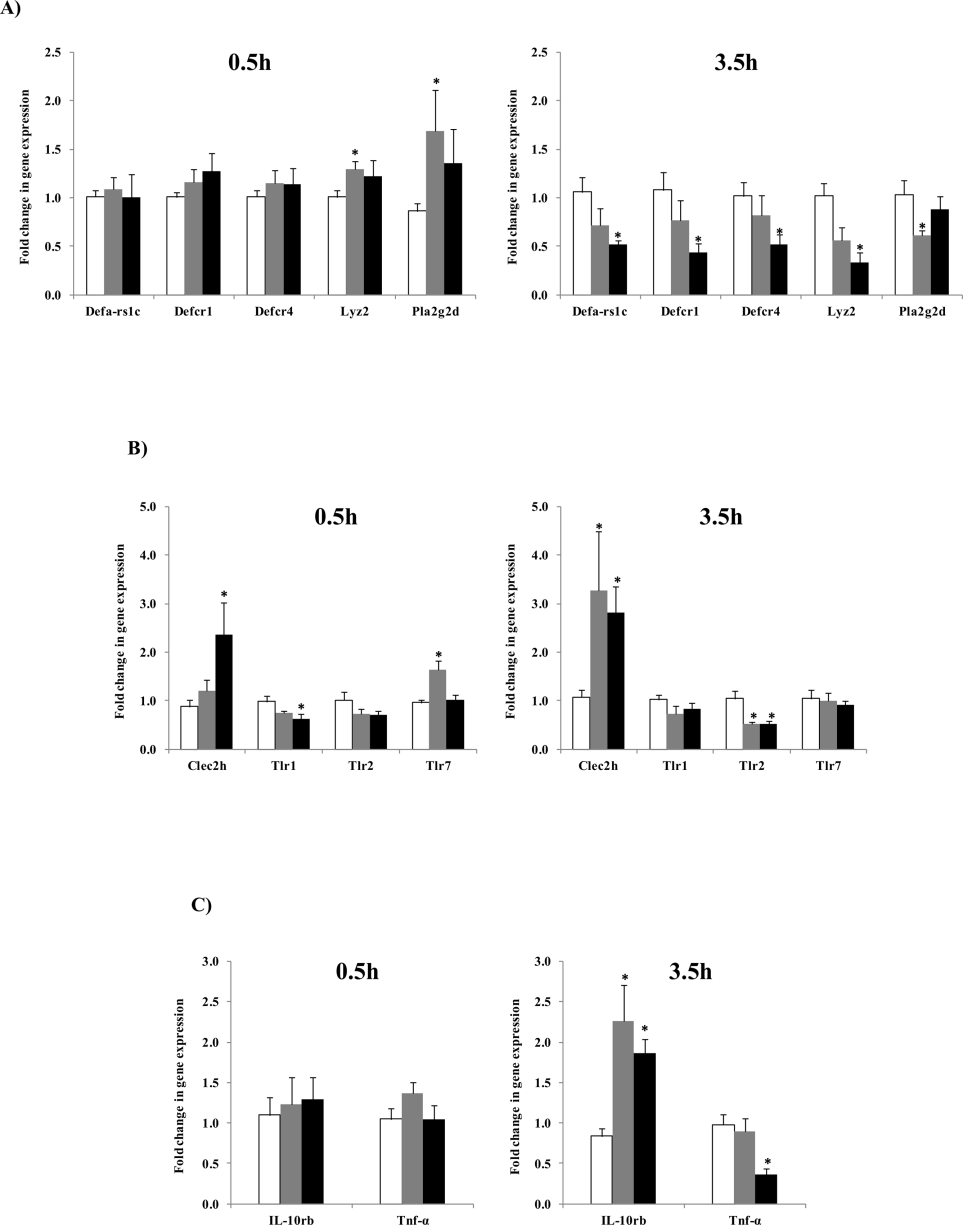
Fold change in gene expression of target genes of the mouse ileum in the control group (white bar) and *L*. *casei* 32G groups; Low dose, 10^6^ CFU/ mouse (grey bar) and High dose, 10^8^ CFU/ mouse (black bar) at 0.5h and 3.5h after the last *L*. *casei* 32 administration. (A) Change in AMPs, (B) Change in PRRs, (C) Change in cytokines; * p<0.05: significant differences from the control, (n: 6/group).

The relationship between intestinal microbiota and host immune system functions in two directions. Typically, this interaction is well balanced and a break down in balance can lead to gastrointestinal inflammation and metabolic disorders [60]. Probiotics have been shown to alter the expression of genes involved in host innate immunity [61,62], hence we investigated influence of *L*. *casei* 32G on the innate immune response. Total RNA was isolated from ileal tissue to screen for changes in genes encoding components of the innate immune system resulting from 32G administration. To examine the influence of dose we included mice receiving the low (10^6^ CFU/mouse) and high (10^8^ CFU/mouse) dose of 32G. To examine the influence of time we chose to focus on the 0.5 and 3.5 h time points, as these were the times the 32G bolus reached the cecum (3.5h) and the longest time since the innate immune system had encountered a high level of 32G (0.5h). We targeted 38 genes associated with the innate immune system. Eight of the genes (*IL-12a*, *IL-12b*, *IL-12rb1*, *IL-12rb2*, *IL-22*, *Pla2g1b*, *Ifng*, and *TLR6*) were below the limit of detection in all samples. Eighteen of the targeted genes (*Lyz1-2*, *Pla2g2*, *sPLA2-IIA*, *Occludin*, *ZO-1*, *IL-10ra*, *Lyz1*, *Nf-kB1*, *Pla2g10*, *Pla2g12a*, *Reg3β*, *Reg3γ*, *Tgfβ1*, *TLR3*, *TLR4*, *TLR5*, *TLR8 and TLR9*) showed no statistical differences from the control in all samples (data not shown). Twelve of the targeted genes (*Defa-rs1c*, *Defcr1*, *Defcr4*, *Lyz2*, *Pla2g2d*, *Clec2h*, *Tlr1*, *Tlr2*, *Tlr7*, *IL-10rb*, *Tnf-α*, and *ZO-2*) were expressed statistically (p<0.05) different from the control mice in some samples and are presented in Fig 4.

Intestinal cells sense microbial ligands through pattern recognition receptors (PRRs) that contribute to cross-talk between the gut microbiota and the innate immune system [63]. We screened intestinal PRRs including Toll-like receptors (TLRs) and C-type lectin-like receptor 2h (Clec2h) to evaluate the effect of 32G on PRRs. Levels of TLR3, 4, 5, 8 and 9 expression were not significantly different than that observed in the control mice at any of the doses or time points examined. TLR6 expression was lower than the limit of detection at all doses and times examined. Alterations were observed in the expression of Clec2h, TLR1, TLR2, and TLR7 (Fig 4). The discussion of these results will focus on Clec2h and TLR2, as consistent significant (p<0.05) results were observed. TLR2 is a receptor that recognizes Gram (+) bacteria [58,64]. TLR2 was lower at the 3.5h time point in mice fed the 10^6^ and 10^8^ CFU/mouse doses, while no significant differences were observed at the 0.5h time point. A similar result was observed in an *in vitro* study where a vaginal epithelial cell line subjected to *Lactobacillus* strains (*L*. *rhamnosus* GR-1W and *L*. *reuteri* RC-14). The epithelial cells had a reduction in TLR2 expression compared to control [65]. The authors hypothesized that exposure to elevated of Gram (+) bacteria might create a state of hyporesponsiveness and results in a decrease in TLR2 expression [66]. Another PRR we targeted, Clec2h (C-type lectin receptor), was significantly (p < 0.05) up-regulated in the high dose at 0.5h and in both doses at the 3.5h time point. The function of Clec2h is poorly defined; however, it is thought to have a role in regulating innate immune responses [67]. These results suggest that administration of 32G alters the expression of genes known to be involved in the regulation of the innate immune system.

Antimicrobial peptides (AMPs) are components of innate immune system that are active in intestinal mucosal defense and have an important role in shaping the composition of the intestinal microbiota [68,69]. We examined the expression of different AMPs in mouse small intestine to evaluate the influence of *L*. *casei* 32G administration on expression of genes encoding AMPs. Significant (p<0.05) changes were observed in the expression of the alpha defensins (Defa-rs1c, Defcr1, Defcr4), lysozyme and Pla2g2d (Fig 4A). The discussion of these results will focus upon the alpha defensins and lysozyme as consistent significant (p<0.05) results were observed. Administration of 32G resulted in a dose dependent reduction in expression of alpha defensins and lysozyme-2 at the 3.5h time point, while their expression were not different than control at 0.5h. Salzman et al. showed that paneth cells alpha defensins are essential in homeostatic control regulating and shaping the composition of intestinal microbiota [32]. In addition, Menendez et al. found that alpha defensin gene expression was sensitive to oral antibiotic administration, suggesting that expression of alpha defensins is dependent upon the commensal microbiota [70]. The results suggest that 32G administration resulted in altering the expression of intestinal AMPs, thereby altering the composition the cecal microbiota. Alternatively, AMPs have been shown to be regulated by short-chain fatty acids, such as butyrate, which are produced by microbial fermentation in the gut [71–74]. This explanation is supported by observed significant (p < 0.05) decrease in the *Lachnospiraceae*, a butyrate producing family, in mice microbiota fed with high dose 32G at the 3.5h time point [75]. While our results suggest a role for AMPs in the 32G mediated changes in the composition of the cecal microbiota, other explanations are also possible and further research is required to determine the mechanism(s) by which 32G alters the cecal microbiota.

Cytokines are critical in regulation and development of the innate immune response [76]. Effect of 32G administration on both anti-inflammatory and pro-inflammatory cytokines was analyzed in this study. Of the cytokines examined, significant (p<0.05) changes in expression were only observed with IL10rb and TNF-α. Expression of IL-10rb, an anti-inflammatory marker, was significantly (p<0.05) increased at the 3.5h time point with both doses, relative to the control. In contrast, we observed a significant (p<0.05) reduction in expression of TNF-α, which serves as an indicator of a pro-inflammatory response, with the high dose at the 3.5h [10]. IL-10rb is a receptor shared by IL-10 subfamily; it is required for the activation of the IL-10 subfamily and a deficiency in IL-10rb has been shown to result in inflammatory bowel disease [77]. IL-10 regulates the inflammatory response by suppressing the production of pro-inflammatory cytokines such as TNF-α [78]. This is a possible explanation for the reduction of TNF-α expression observed in this study. Another possible explanation for the observed reduction in TNF-α is the reduction in TLR2 expression, as TLR2 expression is positively correlated with TNF-α expression [79,80]. These results suggest that administration of 32G has an anti-inflammatory effect on the murine immune system.

## Conclusion

After administering *L*. *casei* 32G to mice at one of three different doses (10^6^, 10^7^, or 10^8^ CFU/day/mouse) we were able to detect *L*. *casei* 32G by qPCR and monitor its abundance at different time points. We demonstrated that only mice fed a high dose of 32G contained greater than 10^5^ CFU/ml of 32G in their cecal contents and that 32G was present at the highest level 3.5 h after administration. Our data revealed that *L*. *casei* 32G administration was capable of altering murine cecal microbiota and the alteration was dose and time dependent. Additionally, there was a lack of significant differences in the overall microbiota composition, regardless of 32G dose, in all treatments at 12h. These results suggest that the light/dark cycle has a significant (p < 0.05) impact on the compositions of the microbiota, likely due to the level of feed derived-nutrients, and therefore the light/dark cycle must be taken into consideration when designing experiments that follow microbiota composition. *Lactobacillus*, one of the dominant genera, increased in cecum content of mice fed with medium and high dose of *L*. *casei* 32G. We demonstrated that the *Lactobacillus*, which increased in prevalence in the cecal microbiota was not *L*. *casei* 32G. Our results indicate that feeding of *L*. *casei* 32G at 10^7^ and 10^8^ CFU/day/mouse altered the environment, released small peptides or metabolites, present in the cecum such that the growth of commensal lactobacilli was favored. Due to the interaction between gut microbiota and host immunity we examined the effect of 32G on the murine immune system. Interactions between PRRs, AMPs and the gut microbiota result in immune homeostasis [81,82]. The administration of *L*. *casei* 32G alters the gut microbiota composition in a dose and time dependent manner, potentially via changes in the expression of PRRs and AMPs. The 32G induced changes in the gut microbiota result in changes in the expression of cytokines, thereby resulting in an anti-inflammatory modulation of the murine immune system. Future research will evaluate the *L*. *casei* strain specificity for the ability to alter the composition of the murine gut microbiota and modulate immune system.

## Supporting Information

S1 Fig qPCR standard curve for quantification of *L*. *casei* 32G in mouse cecum. The standard curves were generated by amplification of DNA isolated from cecum content spiked with 10^8^, 10^7^, 10^6^, 10^5,^ or 10^4^ CFU/ml of *L*. *casei* 32G culture, (n: 3 for each concentration). A) Standard curve. B) Melting curves.
(PDF)

S2 Fig Alpha rarefaction plots based on Chao1 (A) and Shannon index (B). (PDF)

S3 Fig PCoA plot of weighted UniFrac distances generated from mouse cecum content in the control group and *L*. *casei* 32G groups; 10^6^ CFU/ mouse (low), 10^7^ CFU/ mouse (medium) and 10^8^ CFU/ mouse (high), at 0.5, 3.5, 12, and 24 h after the last administration. Symbols represent data from individual mice, color-coded by the indicated metadata.
(PDF)

S4 Fig Microbial communities of individual mouse cecum content at genus level in the control and *L*. *casei* 32G groups; 10^6^ CFU/ mouse (low), 10^7^ CFU/ mouse (medium) and 10^8^ CFU/ mouse (high). (PDF)

S5 Fig Comparison of predominant genera in the microbiota of mice cecums sorted based on dose. The major microbial communities of mouse cecum content at genus level in the control group and *L*. *casei* 32G groups; 10^6^ CFU/ mouse (low), 10^7^ CFU/ mouse (medium) and 10^8^ CFU/ mouse (high), at 0.5, 3.5, 12, and 24 h after the last administration. Only genera with over 5% of the total bacteria are presented (n: 6 for each bar).
(PDF)

S1 Table Expression profile of Lactobacillus casei 32G gene at 3.5h time point. (PDF)

S2 Table Bacterial genera detected^a^ in cecum content of mice administered saline (control) or *Lactobacillus casei* 32G once per day by oral gavage sorted based on dose. (PDF)

S3 Table pH of mouse cecum content after sacrificing. (PDF)
