# Supplementary material for: The Effect of Lactobacillus casei 32G on the Mouse Cecum Microbiota and Innate Immune Response Is Dose and Time Dependent
Source: PLoS One. 2015 Dec 29;10(12):e0145784. doi: 10.1371/journal.pone.0145784 (PMC4705108; doi:10.1371/journal.pone.0145784)
Supplement: S1 Fig — The standard curves were generated by amplification of DNA isolated from cecum content spiked with 108, 107, 106, 105, or 104 CFU/ml of L. casei 32G culture, (n: 3 for each concentration). A) Standard curve. B) Melting curves. (PDF) [file pone.0145784.s001.pdf]

A)

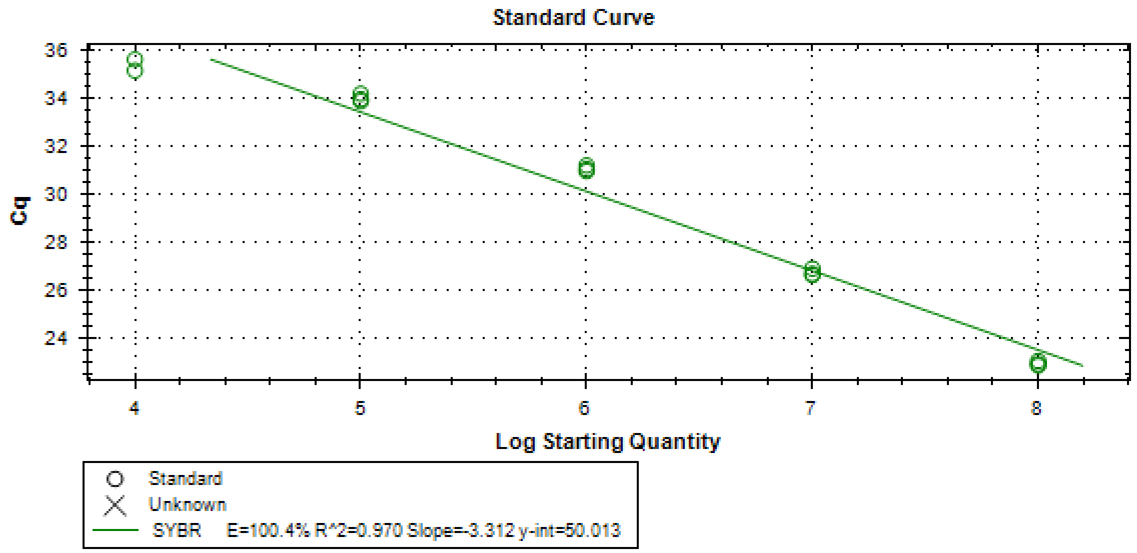

B)

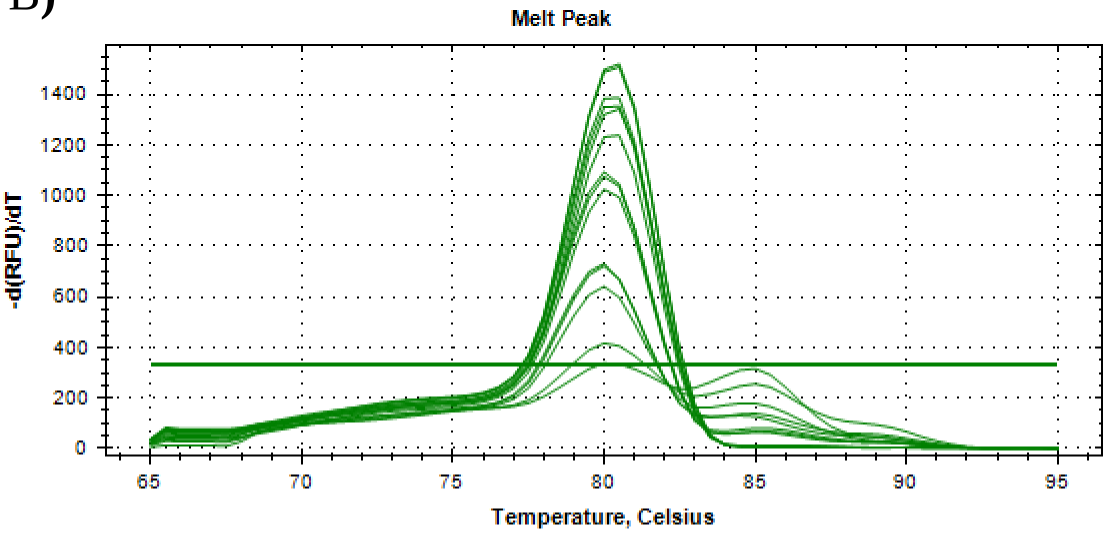

S1 Fig. qPCR standard curve for quantification of *L. casei* 32G in mouse cecum. The standard curves were generated by amplification of DNA isolated from cecum content spiked with 10^8, 10^7, 10^6, 10^5, or 10^4 CFU/ml of *L. casei* 32G culture, (n:3 for each concentration). A) Standard curve. B) Melting curves.
