# Supplementary figures and images for: The Effect of Lactobacillus casei 32G on the Mouse Cecum Microbiota and Innate Immune Response Is Dose and Time Dependent
Source: PLoS One. 2015 Dec 29;10(12):e0145784. doi: 10.1371/journal.pone.0145784 (PMC4705108; doi:10.1371/journal.pone.0145784)

**A)**

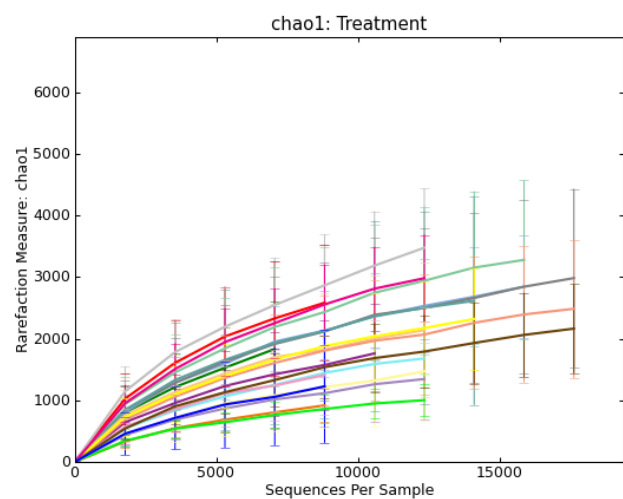

**B)**

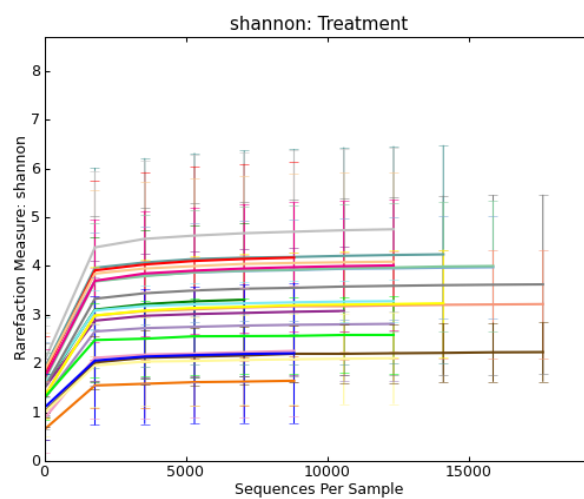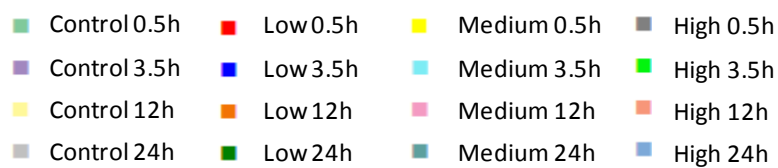

**S2 Fig.** Alpha rarefaction plots based on Chao1 (A) and Shannon index (B).

Supplement: S2 Fig — (PDF) [file pone.0145784.s002.pdf]
