# Supplementary material for: The Effect of Lactobacillus casei 32G on the Mouse Cecum Microbiota and Innate Immune Response Is Dose and Time Dependent
Source: PLoS One. 2015 Dec 29;10(12):e0145784. doi: 10.1371/journal.pone.0145784 (PMC4705108; doi:10.1371/journal.pone.0145784)
Supplement: S3 Fig — Symbols represent data from individual mice, color-coded by the indicated metadata. (PDF) [file pone.0145784.s003.pdf]

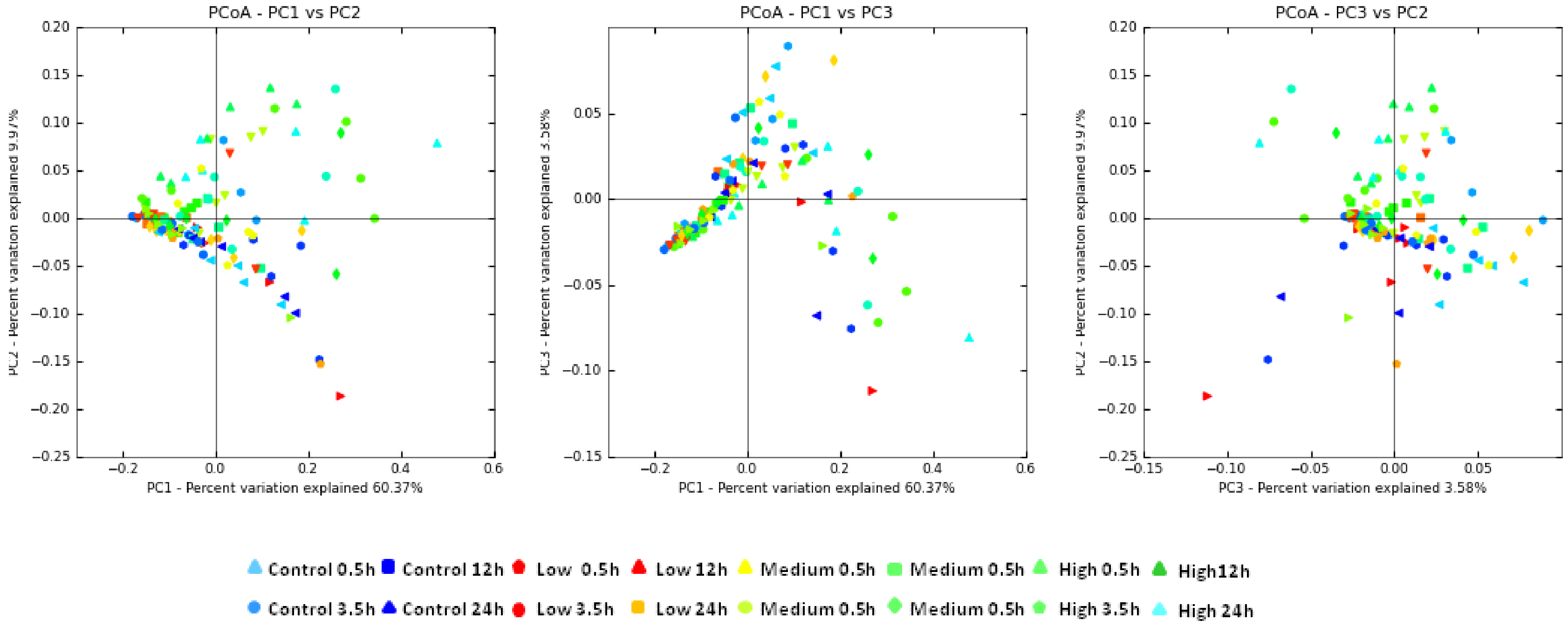

S3 Fig. PCoA plot of weighted UniFrac distances generated from mouse cecum content in the control group and *L. casei* 32G groups; 10^6 CFU/ mouse (low), 10^7 CFU/ mouse (medium) and 10^8 CFU/ mouse (high), at 0.5, 3.5, 12, and 24 h after the last administration. Symbols represent data from individual mice, color-coded by the indicated metadata.
