# Supplementary material for: The Effect of Lactobacillus casei 32G on the Mouse Cecum Microbiota and Innate Immune Response Is Dose and Time Dependent
Source: PLoS One. 2015 Dec 29;10(12):e0145784. doi: 10.1371/journal.pone.0145784 (PMC4705108; doi:10.1371/journal.pone.0145784)
Supplement: S5 Fig — The major microbial communities of mouse cecum content at genus level in the control group and L. casei 32G groups; 106 CFU/ mouse (low), 107 CFU/ mouse (medium) and 108 CFU/ mouse (high), at 0.5, 3.5, 12, and 24 h after the last administration. Only genera with over 5% of the total bacteria are presented (n: 6 for each bar). (PDF) [file pone.0145784.s005.pdf]

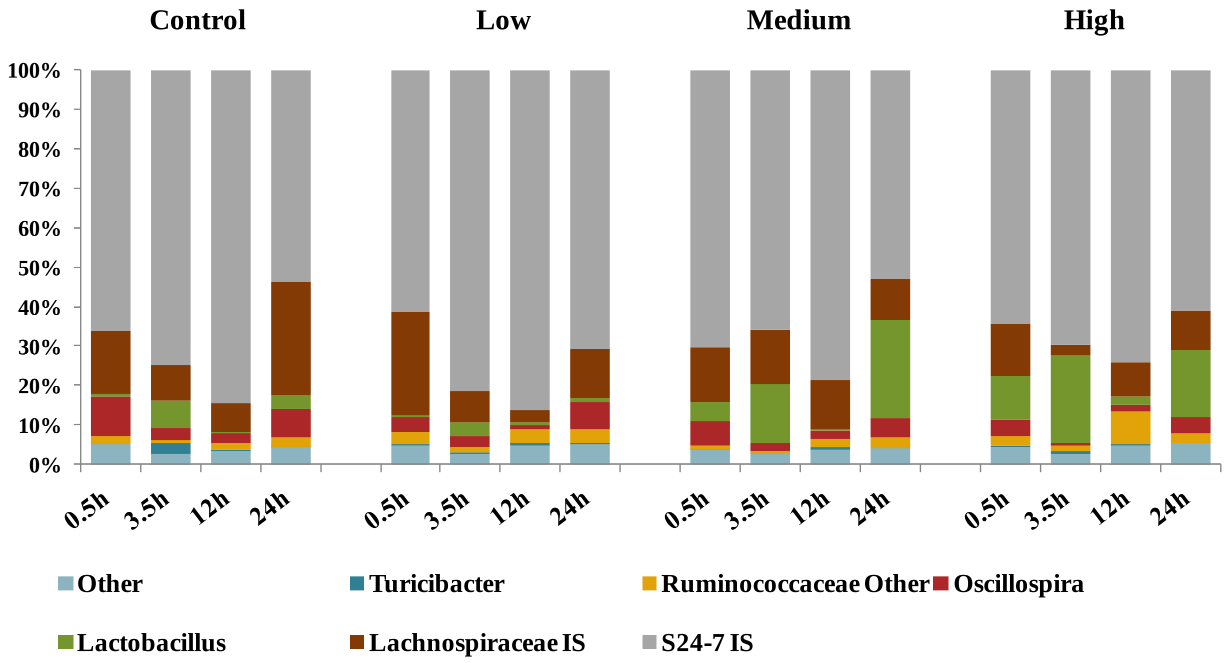

S5 Fig. Comparison of predominant genera in the microbiota of mice cecums sorted based on dose. The major microbial communities of mouse cecum content at genus level in the control group and *L. casei* 32G groups; 10^6 CFU/ mouse (low), 10^7 CFU/ mouse (medium) and 10^8 CFU/ mouse (high), at 0.5, 3.5, 12, and 24 h after the last administration. Only genera with over 5% of the total bacteria are presented (n: 6 for each bar).
