# Supplementary material for: The Effect of Lactobacillus casei 32G on the Mouse Cecum Microbiota and Innate Immune Response Is Dose and Time Dependent
Source: PLoS One. 2015 Dec 29;10(12):e0145784. doi: 10.1371/journal.pone.0145784 (PMC4705108; doi:10.1371/journal.pone.0145784)
Supplement: S1 Table — (PDF) [file pone.0145784.s006.pdf]

S1 Table. Expression profile of *Lactobacillus casei* 32G gene at 3.5h time point.

Mouse Groups	MC properties +/-
Control	–
Control	–
Control	–
Control	–
Low	–
Low	–
Low	–
Low	–
Medium	–
Medium	–
Medium	+
Medium	+
High	+
High	+
High	+
High	+

–, expression not detected; no specific melting curve (MC); +, expression detected
