# Supplementary material for: The Effect of Lactobacillus casei 32G on the Mouse Cecum Microbiota and Innate Immune Response Is Dose and Time Dependent
Source: PLoS One. 2015 Dec 29;10(12):e0145784. doi: 10.1371/journal.pone.0145784 (PMC4705108; doi:10.1371/journal.pone.0145784)
Supplement: S2 Table — (PDF) [file pone.0145784.s007.pdf]

S2 Table. Bacterial genera detected[a] in cecum content of mice administered saline (control) or *Lactobacillus casei* 32G once per day by oral gavage sorted based on dose.

Taxon	Percentage (mean ± SE)[bc]							
	**Control**				**Low**			
	**0.5h**	**3.5h**	**12h**	**24h**	**0.5h**	**3.5h**	**12h**	**24h**
S24-7 IS	66.2 ± 15.2	75.0 ± 15.8	**84.6 ± 10.3**	53.6 ± 22.0	61.4 ± 27.7	81.3 ± 17.5	**86.2 ± 5.9**	70.8 ± 20.7
Lachnospiraceae IS	15.9 ± 9.9	8.9 ± 7.2	**7.1 ± 6.0**	28.8 ± 19.7	26.1 ± 25.0	**8.0 ± 10.2**	**3.3 ± 2.5**	12.3 ± 11.3
Oscillospira	10.0 ± 4.7	**3.3 ± 3.0**	**2.4 ± 2.8**	7.2 ± 3.3	3.8 ± 1.6	2.7 ± 3.6	**1.0 ± 0.6**	7.1 ± 6.2
Ruminococcaceae Other	2.1 ± 1.2	**0.6 ± 0.3**	2.0 ± 1.4	2.3 ± 1.4	3.2 ± 2.3	**1.4 ± 0.6**	3.3 ± 1.9	3.2 ± 1.9
Ruminococcus	1.3 ± 0.6	**0.7 ± 0.4**	1.0 ± 0.5	0.8 ± 0.3	1.1 ± 0.7	**0.5 ± 0.4**	1.9 ± 1.4	1.0 ± 0.3
Clostridia Other	1.2 ± 0.8	**0.3 ± 0.1**	**0.5 ± 0.5**	0.9 ± 0.3	1.0 ± 0.6	0.6 ± 0.6	0.6 ± 0.3	0.8 ± 1.2
Lactobacillus	0.9 ± 1.6	**6.8 ± 7.7**	0.4 ± 0.5	3.7 ± 4.3	0.5 ± 0.9	3.6 ± 6.9	0.6 ± 0.9	1.1 ± 1.0
Lachnospiraceae Other	0.9 ± 0.4	**0.3 ± 0.2**	**0.4 ± 0.5**	0.8 ± 0.4	0.9 ± 0.5	**0.3 ± 0.4**	0.7 ± 0.8	1.1 ± 1.5
Clostridiales Other	0.6 ± 0.3	**0.1 ± 0.1**	**0.3 ± 0.3**	0.6 ± 0.3	0.6 ± 0.4	**0.3 ± 0.3**	**0.2 ± 0.1**	0.5 ± 0.3
Ruminococcaceae IS	0.2 ± 0.1	0.3 ± 0.2	0.2 ± 0.1	0.2 ± 0.1	0.3 ± 0.4	0.2 ± 0.3	0.6 ± 0.7	0.4 ± 0.2
Firmicutes Other	0.2 ± 0.1	0.2 ± 0.1	0.2 ± 0.2	0.2 ± 0.1	0.1 ± 0.2	0.2 ± 0.2	0.2 ± 0.1	**0.4 ± 0.3**
Bacteria Other	0.2 ± 0.0	0.2 ± 0.1	0.2 ± 0.1	**0.2 ± 0.1**	0.1 ± 0.1	0.2 ± 0.1	0.1 ± 0.0	**0.2 ± 0.1**
Erysipelotrichaceae IS	0.1 ± 0.1	**0.0 ± 0.0**	**0.1 ± 0.1**	**0.0 ± 0.0**	0.2 ± 0.2	0.1 ± 0.0	0.1 ± 0.1	0.1 ± 0.1
Turicibacter	0.0 ± 0.1	**2.7 ± 2.6**	**0.4 ± 0.5**	0.1 ± 0.1	0.1 ± 0.2	0.2 ± 0.2	**0.8 ± 0.9**	**0.3 ± 0.3**
Coprobacillaceae IS	0.0 ± 0.0	0.0 ± 0.0	0.0 ± 0.0	0.0 ± 0.0	0.0 ± 0.0	**0.1 ± 0.1**	**0.0 ± 0.0**	**0.2 ± 0.2**
Adlercreutzia	BQL	0.2 ± 0.5	0.1 ± 0.2	0.1 ± 0.0	0.1 ± 0.1	0.1 ± 0.2	0.1 ± 0.2	0.2 ± 0.3
Coriobacteriales IS	BQL	0.0 ± 0.0	BQL	0.0 ± 0.0	BQL	BQL	BQL	0.0 ± 0.0

[a]Only genera that were present at ≥1% in a sample are included in this table.
[b]The detection limit was 0.00003 and this value was used to calculate the p-value.
[c]Genera that differ from 0.5 h time point within each group are shown in bold ($p \leq 0.05$).
IS: Incertae Sedis.
BQL: Below quantifiable limit.

Table S1. ***(Continued)***

Taxon	Percentage (mean ± SE)[bc]							
	**Medium**				**High**			
	**0.5h**	**3.5h**	**12h**	**24h**	**0.5h**	**3.5h**	**12h**	**24h**
S24-7 IS	70.4 ± 13.0	65.9 ± 5.9	78.8 ± 20.3	52.9 ± 31.2	64.3 ± 25.3	69.7 ± 15.6	74.1 ± 14.0	60.9 ± 18.1
Lachnospiraceae IS	13.8 ± 9.6	13.9 ± 8.3	12.2 ± 14.6	10.7 ± 12.2	13.2 ± 8.5	**2.7 ± 1.6**	8.8 ± 8.4	10.2 ± 6.7
Oscillospira	5.9 ± 3.9	**2.0 ± 1.3**	**2.1 ± 2.7**	4.9 ± 4.9	4.3 ± 4.1	**0.8 ± 0.6**	1.9 ± 1.6	4.2 ± 3.1
Ruminococcaceae Other	1.2 ± 0.8	**0.5 ± 0.2**	2.0 ± 2.1	2.6 ± 2.5	2.4 ± 1.9	1.5 ± 1.0	**8.3 ± 4.1**	2.2 ± 0.9
Ruminococcus	0.4 ± 0.2	**0.2 ± 0.1**	0.7 ± 0.8	0.6 ± 0.5	1.2 ± 1.4	0.7 ± 0.8	1.2 ± 0.7	1.4 ± 1.2
Clostridia Other	0.4 ± 0.4	0.2 ± 0.1	0.3 ± 0.2	0.7 ± 0.7	0.7 ± 0.7	**0.2 ± 0.1**	0.6 ± 0.5	0.8 ± 0.6
Lactobacillus	5.0 ± 4.6	**15.0 ± 9.6**	**0.6 ± 0.4**	**24.8 ± 17.3**	11.0 ± 16.4	22.2 ± 14.6	1.9 ± 2.7	16.9 ± 19.5
Lachnospiraceae Other	0.4 ± 0.2	**0.2 ± 0.1**	0.3 ± 0.2	0.5 ± 0.5	0.7 ± 0.6	**0.2 ± 0.1**	0.6 ± 0.5	0.8 ± 0.4
Clostridiales Other	0.5 ± 0.4	**0.2 ± 0.1**	0.3 ± 0.2	0.6 ± 0.8	0.4 ± 0.4	**0.1 ± 0.1**	0.6 ± 0.5	0.4 ± 0.2
Ruminococcaceae IS	0.9 ± 0.6	0.6 ± 0.3	1.0 ± 0.6	**0.4 ± 0.3**	0.7 ± 0.9	0.8 ± 0.4	0.8 ± 0.5	0.5 ± 0.2
Firmicutes Other	0.2 ± 0.1	0.2 ± 0.1	0.2 ± 0.1	**0.4 ± 0.2**	0.2 ± 0.2	0.2 ± 0.1	0.1 ± 0.1	0.4 ± 0.2
Bacteria Other	0.3 ± 0.1	0.4 ± 0.2	**0.2 ± 0.1**	0.3 ± 0.1	0.2 ± 0.1	0.3 ± 0.1	0.4 ± 0.2	**0.5 ± 0.1**
Erysipelotrichaceae IS	0.1 ± 0.1	0.1 ± 0.1	0.2 ± 0.2	0.1 ± 0.0	0.0 ± 0.0	0.0 ± 0.0	0.0 ± 0.0	0.1 ± 0.0
Turicibacter	0.0 ± 0.0	**0.1 ± 0.2**	**0.8 ± 1.1**	0.0 ± 0.0	0.3 ± 0.4	0.4 ± 0.7	0.4 ± 0.5	**0.0 ± 0.1**
Coprobacillaceae IS	0.0 ± 0.0	**0.0 ± 0.0**	0.0 ± 0.0	0.0 ± 0.0	0.0 ± 0.0	0.0 ± 0.0	0.0 ± 0.0	**0.0 ± 0.0**
Adlercreutzia	0.2 ± 0.1	0.3 ± 0.2	0.2 ± 0.1	0.2 ± 0.2	0.0 ± 0.0	**0.1 ± 0.0**	0.0 ± 0.0	**0.1 ± 0.1**
Coriobacteriales IS	BQL	BQL	BQL	BQL	BQL	BQL	BQL	**0.2 ± 0.2**

[a]Only genera that were present at ≥1% in a sample are included in this table, the complete data set is presented in Table SX.
[b]The detection limit was 0.00003 and this value was used to calculate the p-value.
[c]Genera that differ from 0.5 h time point within each group are shown in bold (p≤0.05).
IS: Incertae Sedis.
BQL: Below quantifiable limit.
