# Supplementary material for: The Effect of Lactobacillus casei 32G on the Mouse Cecum Microbiota and Innate Immune Response Is Dose and Time Dependent
Source: PLoS One. 2015 Dec 29;10(12):e0145784. doi: 10.1371/journal.pone.0145784 (PMC4705108; doi:10.1371/journal.pone.0145784)
Supplement: S3 Table — (PDF) [file pone.0145784.s008.pdf]

S3 Table. pH of mouse cecum content after sacrificing.

	**pH (mean ± SE)***			
	**0.5h**	**3.5h**	**12h**	**24h**
Control	7.2 ± 0.2	7.6 ± 0.2	7.1 ± 0.0	7.3 ± 0.1
Low	7.3 ± 0.2	7.8 ± 0.1	7.1 ± 0.1	7.5 ± 0.2
Medium	**7.6 ± 0.1**	7.7 ± 0.1	7.1 ± 0.1	**7.6 ± 0.1**
High	**7.5 ± 0.2**	7.6 ± 0.1	7.1 ± 0.1	7.4 ± 0.1

*pH that differs from control within each group is shown in bold ($p \leq 0.05$).
